# Objective Clinical and Radiological Response under Sunitinib in a Case of Thigh Hidradenocarcinoma

**DOI:** 10.1155/2020/9656475

**Published:** 2020-02-25

**Authors:** S. Korbi, H. Rachdi, H. El Benna, N. Mejri, Y. Berrazaga, N. Daoud, S. Labidi, H. Boussen

**Affiliations:** Medical Oncology Department, University Hospital Abderrahman Mami, Ariana, Boulevard de l'Hopital, 2080 Tunis, Tunisia

## Abstract

A 56-year-old male was treated by local surgery in 1968 and 2005 for a left thigh lesion. A 2^nd^ local relapse occurred in 2015 and was treated by complete macroscopic surgery with histology concluding to a hidradenocarcinoma. A 3^rd^ locoregional relapse occurred in October 2018, with the presence of inflammatory ulcerated lesions. A 2^nd^ histology and immunohistochemistry exam showed a proliferation positive for CK, CK5, and p63 suggesting the diagnosis of hidradenocarcinoma. The patient was treated by 3 lines of chemotherapy, 1^st^ by Adriamycin, 2^nd^ by carboplatin-paclitaxel, and then 3^rd^ by oral capecitabine, leading to a stable clinical disease but without a clinical benefit. A locoregional plus metastatic lung progression was observed in March 2019, with the presence of lung nodules and retroperitoneal lymph nodes, multiple skin left thigh and left inguinal ulcerated lesions. The patient received then in 4^th^ line in April 2019 oral sunitinib at 50 mg daily, with 4 weeks therapy/2 weeks pause. Side effects were represented by mucositis, anorexia, weight loss, and fatigue. We observed since the 1^st^ week of therapy a fast response, with a decrease of the ulcerated lesions, a skin loss, and deep hemorrhagic areas. CT-scan showed after 2 weeks of sunitinib an objective response on both locoregional and metastatic lesions.

## 1. Introduction

Hidradenocarcinoma (Hidrad) is a rare skin adnexal malignancy, arising from sweat structures, representing 6% of malignant eccrine tumors and less than 0.001% of all cancers [1, 2]. Like for other skin cancers, standard treatment is local surgery, and in relapsing patients, few responses have been reported under salvage chemotherapy or targeted therapies [3–5]. We report herein an observation of impressive clinical and radiological response under sunitinib in a relapsing metastatic case.

## 2. Observation

**Figure 1:**
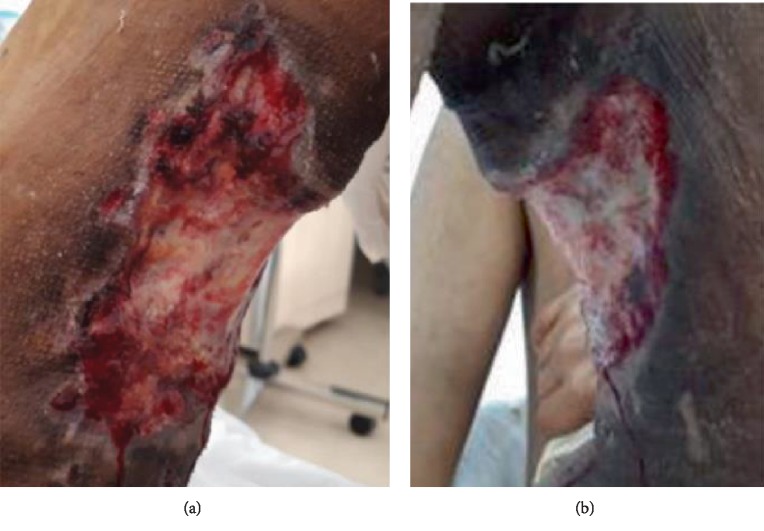
(a, b) Left limb ulcerated lesions, before and after 2 months of sunitinib, with pigmentation, ulceration decrease, and skin loss in the responding area.

**Figure 2:**
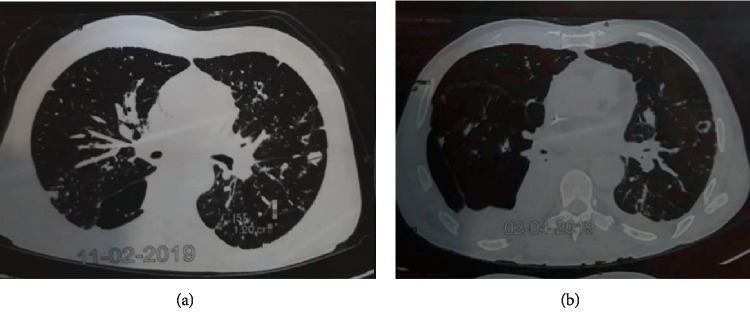
(a, b) Response on left lung nodules after 2 months of sunitinib.

A 56-year-old patient was treated by local surgery for a left thigh lesion operated in 1968 and 2005. A 2^nd^ local relapse was observed 10 years later in 2015 and treated by local resection. A 3^rd^ local and inguinal relapse occurred in October 2018 with the presence of multiple red inflammatory and ulcerated left thigh and inguinal lesions. Histologic plus immunohistochemistry exam showed a proliferation positive for CK, CK5, and p63, suggesting the diagnosis of hidradenocarcinoma. He received 3 lines of chemotherapy with Adriamycin alone, carboplatin-paclitaxel, and then oral capecitabine with stable disease and no clinical benefit. Due to metastatic progression to lungs under capecitabine, we started in March 2019 a 4^th^ line with oral sunitinib at 50 mg daily dose, 4 weeks treatment, and 2 weeks off. We observed a fast clinical response after two months of treatment on left lower limb edema as well as inguinal and left thigh lesion (Figures 1(a) and 1(b)). Radiologic evaluation by CT-scan showed an objective response on left thigh and inguinal lesions as well as metastatic lung nodules (Figures 2(a) and 2(b)).

## 3. Discussion

We report a new case of Hidrad in a 56-year-old man, initially resected in 1968 with local relapses in 2005 and 2015 and metastatic failure in 2018. This observation is particular by a long evolution between the probable hidradenoma “status” and the late relapse at the hidradenocarcinoma “phase,” with a gap of 37 years. Hidrad usually affects male adults between sixth and seventh decade, as we observed for our case [1–3]. This aggressive skin adnexal tumor of sweat glands also called clear cell eccrine carcinoma, malignant nodular hidradenoma or malignant clear cell hidradenoma, remains exceptional representing less than 0.001% of all cancers [1–3]. Hidrad occurs as “de novo” tumor or mostly within a preexisting hidradenoma like for our patient, operated in 1968 for a left thigh lesion and relapsing in 2005 and 2015, when diagnosis was confirmed [3, 4]. It occurs mostly in the head and neck area and particularly the face or more rarely lower limbs or groin, like left thigh in our case [1–3]. Clinical aspect is usually in the form of unique or multiple firm, subcutaneous nodules, or erythematous plaque that could be ulcerated, like for our patient [1–3]. Conventional morphology identified two different cell types of Hidrad, eosinophilic cytoplasm laiden darker fusiform/spindle cells or large clear cells exhibiting atypical mitotic figures and nuclear pleomorphism [4]. IHC is usually positive for CK, EMA (epithelial membrane antigen), CEA (carcinoembryonic antigen), and S100 protein, and our case expressed on IHC a positivity for CK, CK7, and p63 [4]. Usual treatment for Hidrad is wide local excision, with or without lymph node dissection [5–7]. Adjuvant and/or palliative radiation therapy is rarely used [7]. Salvage chemotherapy in locoregional and/or metastatic relapse showed a modest efficacy, like we observed for our patient that received 3 lines of treatment [7]. After metastatic progression, we opted for a 4^th^ line oral sunitinib, an oral tyrosine kinase inhibitor having an antiangiogenic and antitumor activity. This drug acts by inhibiting vascular endothelial growth factor receptor (VEGFR), platelet-derived growth factor receptor (PDGFR), c-KIT, FLT3, CSF-1, and RET, usually indicated in advanced renal cell carcinoma, and imatinib-refractory GISTs, but also neuroendocrine and thyroid cancers [8, 9]. Surprisingly in this skin adnexal tumor, we observed an impressive clinical and radiological fast response starting after a week of administration of sunitinib. We collected 2 similar exceptional cases of Hidrad patients, responders to sunitinib in 3^rd^ and 2^nd^ line after salvage chemotherapy [8]. They reported an impressive and durable response on skin lesions, like for our patient. For our part, we are impressed by the rapid response after a week of treatment on the skin lesions that decreased and disappeared with a parallel extended skin loss and occurrence of hemorrhagic areas probably related to the antiangiogenic effect of sunitinib. This fast response could be explained by the presence of a high level of VEGF in this tumor type. In one particular case of Hidrad mimicking pyogenic granuloma, Mitamura et al. observed a high level of VEGF, induced by repeated injury to the tumoral stroma. Their case was positive for keratin AE1/3, cytokeratin 7, carcinoembryonic antigen, epithelial membrane antigen, and p53 [9]. Their case presented with an upper exophytic lesion containing a markedly edematous stroma with a mixed cellular infiltrate (lymphocytes, CD68+ macrophages, and neutrophils) and plenty of capillaries. The endothelial cells, stromal macrophage-like cells, and extracellular matrix surrounding the capillaries were highly positive for vascular endothelial growth factor (VEGF). They hypothesize that VEGF production may induce capillary formation and fast growing of the tumor, and this could partially explain the dramatic response that we observed for our part, on the multiple left thigh and inguinal tumors, but also the normal cutaneous and subcutaneous tissue close to the tumor, inducing a large and extended abrasion probably by the antiangiogenic effect of sunitinib. We think that despite the fact that Hidrad is exceptional, oral TKI's like sunitinib could be a good option with salvage intent. Therapeutic innovations like immunotherapy or targeted therapies could be indicated after progression under chemotherapy for rare tumors, like Merkel cell carcinoma [10].

## 4. Conclusion

Hidradenocarcinoma remains a rare but aggressive entity. Its diagnosis is based on clinicopathological confrontation. Additional clinical researches to guide treatment decisions are needed.
